# *n*-Butyl­dichlorido{4-cyclo­hexyl-1-[phenyl(2-pyridyl-κ*N*)methyl­ene]­thiosemicarbazidato-κ^2^ *N* ^1^,*S*}tin(IV) chloro­form monosolvate

**DOI:** 10.1107/S1600536810014455

**Published:** 2010-04-24

**Authors:** Md. Abu Affan, Md. Abdus Salam, Yang Farina, Seik Weng Ng

**Affiliations:** a Faculty of Resource Science and Technology, Universiti Malaysia Sarawak, 94300 Kota Samarahan, Sarawak, Malaysia; b School of Chemical Sciences and Food Technology, Universiti Kebangsaan Malaysia, 43600 Bangi, Malaysia; c Department of Chemistry, University of Malaya, 50603 Kuala Lumpur, Malaysia

## Abstract

The monodeprotonated Schiff base ligand in the title com­pound, [Sn(C_4_H_9_)(C_19_H_21_N_4_S)Cl_2_]·CHCl_3_, *N*,*N*′,*S*-chelates to the Sn atom, which is six-coordinated in an octa­hedral environment. The three coordinating atoms along with the butyl C atom comprise a square plane, above and below which are positioned the Cl atoms. The amino group is a hydrogen-bond donor to a Cl atom of an adjacent mol­ecule, the hydrogen bond giving rise to a helical chain propagating in [010]. The Cl and H atoms of the chloro­form mol­ecule are disordered over two positions in an 0.67:0.33 ratio.

## Related literature

For the crystal structures of other metal derivatives of the Schiff base, see: Joseph *et al.* (2004 ▶).
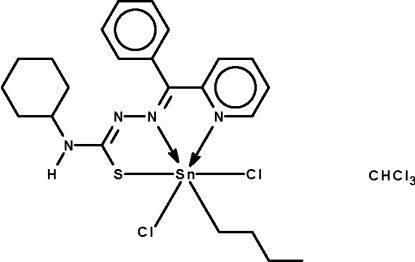


## Experimental

### 

#### Crystal data

[Sn(C_4_H_9_)(C_19_H_21_N_4_S)Cl_2_]·CHCl_3_
*M* *_r_* = 703.53
Monoclinic, 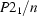

*a* = 14.5095 (9) Å
*b* = 13.7308 (8) Å
*c* = 15.7412 (10) Å
β = 94.418 (1)°
*V* = 3126.8 (3) Å^3^
*Z* = 4
Mo *K*α radiation
μ = 1.33 mm^−1^
*T* = 293 K
0.40 × 0.30 × 0.20 mm

#### Data collection

Bruker SMART APEX diffractometer
Absorption correction: multi-scan (*SADABS*; Sheldrick, 1996 ▶) *T*_min_ = 0.618, *T*_max_ = 0.777
29403 measured reflections
7179 independent reflections
5127 reflections with *I* > 2σ(*I*)
*R*_int_ = 0.032

#### Refinement

*R*[*F* ^2^ > 2σ(*F* ^2^)] = 0.058
*wR*(*F* ^2^) = 0.202
*S* = 1.10
7179 reflections
332 parameters
105 restraints
H-atom parameters constrained
Δρ_max_ = 1.48 e Å^−3^
Δρ_min_ = −1.14 e Å^−3^

### 

Data collection: *APEX2* (Bruker, 2009 ▶); cell refinement: *SAINT* (Bruker, 2009 ▶); data reduction: *SAINT*; program(s) used to solve structure: *SHELXS97* (Sheldrick, 2008 ▶); program(s) used to refine structure: *SHELXL97* (Sheldrick, 2008 ▶); molecular graphics: *X-SEED* (Barbour, 2001 ▶); software used to prepare material for publication: *publCIF* (Westrip, 2010 ▶).

## Supplementary Material

Crystal structure: contains datablocks global, I. DOI: 10.1107/S1600536810014455/bt5251sup1.cif

Structure factors: contains datablocks I. DOI: 10.1107/S1600536810014455/bt5251Isup2.hkl

Additional supplementary materials:  crystallographic information; 3D view; checkCIF report

## Figures and Tables

**Table 1:** Selected bond lengths (Å)

Sn1—C1	2.142 (7)
Sn1—N1	2.250 (5)
Sn1—N2	2.221 (5)
Sn1—S1	2.475 (2)
Sn1—Cl1	2.515 (2)
Sn1—Cl2	2.496 (2)

**Table 2:** Hydrogen-bond geometry (Å, °)

*D*—H⋯*A*	*D*—H	H⋯*A*	*D*⋯*A*	*D*—H⋯*A*
N4—H4⋯Cl1^i^	0.86	2.54	3.383 (6)	167

Symmetry code: (i) 
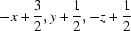
.

**Fig. 1.**
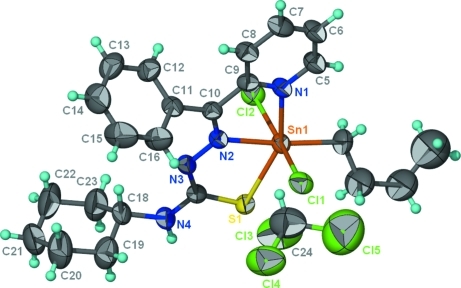
Anisotropic displacement ellipsoid plot (Barbour, 2001) of SnCl2(C4H9)(C19H21N4S).CHCl3 at the 50% probability level; hydrogen atoms are drawn as spheres of arbitrary radius. The disorder in the chloroform molecule is not shown.

## supplementary crystallographic information

### Comment

The mono-deprotonated anion of 2-benzoylpyridine 4-cyclohexyl thiosemicarbazone is a ligand that *N*,*N*',*S*-binds to metal atoms (Joseph *et al.*, 2004). Whereas similar ligands have been complexed with diorganotin and triorganotin systems, the monoorganotin analogs have not been so extensively studied. The mono-deprotonated Schiff-base ligand in SnCl_2_(C_4_H_9_)(C_19_H_12_N_4_S)^.^CHCl_3_*N*,*N*',*S*-chelates to the tin atom, which is six-coordinate in an octahedral environment (Scheme I, Fig. 1). The three coordinating atoms along with the *ipso*-butyl carbon comprise a square plane, above and below which are positioned the chlorine atoms.

### Experimental

2-Benzoylpyridine 4-cyclohexyl thiosemicarbazone was synthesized by using a literature method (Joseph *et al.*, 2004). The compound (0.34 g, 1 mmol) was dissolved in dry methanol (10 ml) in a Schlenk apparatus under a nitrogen atmosphere. *n*-Butyltin trichoride (0.28 g, 1 mmol) dissolved in methanol (10 ml) was added. The mixture was heated for an hour. The solvent was removed and the yellow compound recrystallized from chloroform/methanol (1:1) in 70% yield, m.p. 478-480 K.

### Refinement

Hydrogen atoms were placed in calculated positions (C—H 0.93 to 0.97, N–H 0.86 Å) and were included in the refinement in the riding model approximation, with *U*(H) set to 1.2 to 1.5*U*_eq_(C).

For the butyl chain and cyclohexyl ring, the 1,2 carbon-carbon distances were restrained to 1.54±0.01 Å and the 1,3  ones to 2.51±0.01 Å. The anisotropic displacement ellipsoids of the C_β_, C_γ_ and C_δ_ atoms of the chain were restrained to be nearly isotropic.

The solvent molecule is disodered over two positions. The occupancy could not be refined, and was estimated by an examination of their temperature factors to be a 2:1 disorder. The six carbon-chlorine distances were restrained to within 0.01 Å of each other, as were the pairs of chlorine-chlorine distances. The anisotropic temperature factors were similar restrained to be nearly isotropic.

The final difference Fourier map had a peak/hole in the vicinity of the solvent molecule.

### Crystal data

**Table:**

[Sn(C_4_H_9_)(C_19_H_21_N_4_S)Cl_2_]·CHCl_3_	*F*(000) = 1416
*M**_r_* = 703.53	*D*_x_ = 1.495 Mg m^−^^3^
Monoclinic, *P*2_1_/*n*	Mo *K*α radiation, λ = 0.71073 Å
Hall symbol: -P 2yn	Cell parameters from 8312 reflections
*a* = 14.5095 (9) Å	θ = 2.3–24.6°
*b* = 13.7308 (8) Å	µ = 1.33 mm^−^^1^
*c* = 15.7412 (10) Å	*T* = 293 K
β = 94.418 (1)°	Prism, orange
*V* = 3126.8 (3) Å^3^	0.40 × 0.30 × 0.20 mm
*Z* = 4	

### Data collection

**Table:**

Bruker SMART APEX diffractometer	7179 independent reflections
Radiation source: fine-focus sealed tube	5127 reflections with *I* > 2σ(*I*)
graphite	*R*_int_ = 0.032
ω scans	θ_max_ = 27.5°, θ_min_ = 1.8°
Absorption correction: multi-scan (*SADABS*; Sheldrick, 1996)	*h* = −18→18
*T*_min_ = 0.618, *T*_max_ = 0.777	*k* = −17→17
29403 measured reflections	*l* = −20→19

### Refinement

**Table:**

Refinement on *F*^2^	Primary atom site location: structure-invariant direct methods
Least-squares matrix: full	Secondary atom site location: difference Fourier map
*R*[*F*^2^ > 2σ(*F*^2^)] = 0.058	Hydrogen site location: inferred from neighbouring sites
*wR*(*F*^2^) = 0.202	H-atom parameters constrained
*S* = 1.10	*w* = 1/[σ^2^(*F*_o_^2^) + (0.1058*P*)^2^ + 5.4806*P*] where *P* = (*F*_o_^2^ + 2*F*_c_^2^)/3
7179 reflections	(Δ/σ)_max_ = 0.001
332 parameters	Δρ_max_ = 1.48 e Å^−^^3^
105 restraints	Δρ_min_ = −1.13 e Å^−^^3^

### Fractional atomic coordinates and isotropic or equivalent isotropic displacement parameters (Å^2^)

**Table:**

	*x*	*y*	*z*	*U*_iso_*/*U*_eq_	Occ. (<1)
Sn1	0.64810 (3)	0.58870 (3)	0.13740 (3)	0.04946 (18)	
Cl1	0.66887 (14)	0.53556 (13)	0.29057 (11)	0.0682 (5)	
Cl2	0.65057 (13)	0.61135 (14)	−0.01975 (11)	0.0670 (4)	
Cl3	0.8024 (6)	0.4670 (4)	0.4790 (4)	0.168 (3)	0.67
Cl4	0.9050 (5)	0.3830 (5)	0.6208 (4)	0.154 (2)	0.67
Cl5	0.7217 (6)	0.3399 (7)	0.5849 (7)	0.251 (4)	0.67
Cl3'	0.7344 (11)	0.4338 (15)	0.5063 (12)	0.225 (9)	0.33
Cl4'	0.9232 (11)	0.4198 (17)	0.4959 (12)	0.282 (11)	0.33
Cl5'	0.8460 (14)	0.3630 (19)	0.6443 (6)	0.271 (13)	0.33
S1	0.70794 (10)	0.75442 (11)	0.16994 (11)	0.0531 (4)	
N1	0.6704 (4)	0.4298 (3)	0.1105 (3)	0.0500 (11)	
N2	0.7996 (3)	0.5648 (4)	0.1381 (3)	0.0458 (10)	
N3	0.8608 (3)	0.6390 (3)	0.1461 (3)	0.0518 (12)	
N4	0.8811 (4)	0.8021 (4)	0.1619 (4)	0.0697 (16)	
H4	0.8588	0.8578	0.1745	0.084*	
C1	0.5004 (5)	0.5961 (6)	0.1334 (6)	0.077 (2)	
H1A	0.4766	0.6069	0.0749	0.092*	
H1B	0.4773	0.5335	0.1509	0.092*	
C2	0.4634 (6)	0.6725 (9)	0.1871 (9)	0.133 (4)	
H2A	0.4890	0.7341	0.1702	0.159*	
H2B	0.4876	0.6604	0.2453	0.159*	
C3	0.3626 (7)	0.6855 (10)	0.1881 (10)	0.152 (5)	
H3A	0.3493	0.6944	0.2471	0.182*	
H3B	0.3470	0.7462	0.1589	0.182*	
C4	0.2979 (9)	0.6108 (13)	0.1514 (13)	0.209 (8)	
H4A	0.2355	0.6335	0.1535	0.314*	
H4B	0.3059	0.5516	0.1836	0.314*	
H4C	0.3101	0.5987	0.0933	0.314*	
C5	0.6025 (5)	0.3647 (5)	0.0968 (5)	0.0636 (17)	
H5	0.5415	0.3860	0.0928	0.076*	
C6	0.6202 (5)	0.2677 (5)	0.0883 (5)	0.0712 (19)	
H6	0.5718	0.2239	0.0774	0.085*	
C7	0.7096 (6)	0.2356 (5)	0.0961 (5)	0.0706 (19)	
H7	0.7228	0.1696	0.0914	0.085*	
C8	0.7794 (5)	0.3018 (4)	0.1109 (4)	0.0576 (15)	
H8	0.8406	0.2810	0.1172	0.069*	
C9	0.7591 (4)	0.3990 (4)	0.1165 (4)	0.0486 (13)	
C10	0.8304 (4)	0.4756 (4)	0.1303 (4)	0.0484 (13)	
C11	0.9298 (2)	0.4507 (3)	0.1343 (3)	0.0560 (15)	
C12	0.9676 (3)	0.4104 (4)	0.0638 (3)	0.0686 (18)	
H12	0.9305	0.3991	0.0140	0.082*	
C13	1.0611 (4)	0.3869 (4)	0.0678 (4)	0.088 (3)	
H13	1.0864	0.3599	0.0207	0.106*	
C14	1.1167 (3)	0.4038 (5)	0.1423 (5)	0.106 (4)	
H14	1.1792	0.3880	0.1450	0.127*	
C15	1.0788 (3)	0.4441 (6)	0.2127 (4)	0.123 (4)	
H15	1.1160	0.4554	0.2625	0.148*	
C16	0.9854 (4)	0.4676 (5)	0.2087 (3)	0.087 (3)	
H16	0.9601	0.4946	0.2559	0.105*	
C17	0.8230 (4)	0.7263 (4)	0.1580 (4)	0.0515 (13)	
C18	0.9787 (4)	0.7986 (5)	0.1466 (5)	0.072 (2)	
H18	0.9957	0.7311	0.1350	0.087*	
C19	1.0359 (5)	0.8338 (9)	0.2262 (5)	0.107 (3)	
H19A	1.0171	0.8994	0.2399	0.128*	
H19B	1.0243	0.7920	0.2739	0.128*	
C20	1.1388 (5)	0.8330 (11)	0.2126 (6)	0.136 (5)	
H20A	1.1731	0.8598	0.2626	0.163*	
H20B	1.1590	0.7663	0.2058	0.163*	
C21	1.1596 (6)	0.8913 (9)	0.1353 (7)	0.121 (5)	
H21A	1.1484	0.9597	0.1460	0.145*	
H21B	1.2245	0.8839	0.1257	0.145*	
C22	1.1017 (6)	0.8603 (10)	0.0562 (6)	0.129 (4)	
H22A	1.1201	0.7955	0.0395	0.155*	
H22B	1.1120	0.9046	0.0099	0.155*	
C23	0.9983 (5)	0.8599 (10)	0.0720 (7)	0.110 (3)	
H23A	0.9783	0.9261	0.0817	0.132*	
H23B	0.9629	0.8355	0.0216	0.132*	
C24	0.8274 (6)	0.3624 (8)	0.5359 (6)	0.134 (4)	
H24	0.8452	0.3083	0.5000	0.161*	0.67
H24'	0.8185	0.2966	0.5127	0.161*	0.33

### Atomic displacement parameters (Å^2^)

**Table:**

	*U*^11^	*U*^22^	*U*^33^	*U*^12^	*U*^13^	*U*^23^
Sn1	0.0436 (3)	0.0431 (3)	0.0616 (3)	−0.00057 (16)	0.00317 (18)	0.00019 (17)
Cl1	0.0875 (12)	0.0550 (9)	0.0624 (10)	−0.0180 (8)	0.0071 (8)	0.0049 (7)
Cl2	0.0722 (11)	0.0693 (10)	0.0584 (9)	−0.0024 (8)	−0.0024 (8)	0.0029 (8)
Cl3	0.247 (7)	0.095 (3)	0.162 (5)	0.047 (4)	0.004 (5)	0.012 (3)
Cl4	0.142 (4)	0.179 (5)	0.132 (4)	0.034 (4)	−0.037 (4)	−0.043 (4)
Cl5	0.218 (7)	0.239 (8)	0.300 (9)	0.027 (7)	0.053 (7)	−0.020 (7)
Cl3'	0.212 (12)	0.233 (12)	0.229 (12)	0.036 (9)	−0.001 (9)	−0.021 (9)
Cl4'	0.286 (14)	0.267 (14)	0.286 (14)	−0.009 (10)	−0.019 (10)	0.005 (10)
Cl5'	0.283 (16)	0.277 (15)	0.253 (15)	−0.022 (10)	0.017 (10)	−0.010 (10)
S1	0.0492 (8)	0.0414 (7)	0.0698 (10)	0.0030 (6)	0.0113 (7)	−0.0050 (7)
N1	0.048 (3)	0.042 (2)	0.059 (3)	−0.004 (2)	−0.003 (2)	0.000 (2)
N2	0.045 (2)	0.042 (2)	0.050 (3)	−0.0014 (19)	0.000 (2)	0.000 (2)
N3	0.046 (3)	0.037 (2)	0.073 (3)	−0.001 (2)	0.008 (2)	−0.008 (2)
N4	0.055 (3)	0.040 (3)	0.116 (5)	−0.004 (2)	0.024 (3)	−0.019 (3)
C1	0.048 (4)	0.092 (6)	0.091 (6)	0.002 (4)	0.008 (4)	−0.011 (4)
C2	0.087 (6)	0.132 (8)	0.179 (9)	0.019 (6)	0.010 (6)	−0.048 (7)
C3	0.112 (7)	0.152 (9)	0.193 (9)	0.022 (7)	0.020 (7)	−0.049 (8)
C4	0.189 (11)	0.206 (12)	0.236 (12)	0.013 (9)	0.029 (9)	−0.030 (9)
C5	0.062 (4)	0.052 (4)	0.075 (4)	−0.006 (3)	−0.002 (3)	−0.002 (3)
C6	0.070 (4)	0.055 (4)	0.087 (5)	−0.018 (3)	−0.001 (4)	−0.004 (4)
C7	0.089 (5)	0.041 (3)	0.081 (5)	−0.008 (3)	−0.001 (4)	−0.002 (3)
C8	0.066 (4)	0.039 (3)	0.067 (4)	0.002 (3)	0.000 (3)	0.001 (3)
C9	0.056 (3)	0.042 (3)	0.048 (3)	0.005 (2)	−0.001 (3)	−0.002 (2)
C10	0.049 (3)	0.040 (3)	0.055 (3)	0.005 (2)	0.001 (2)	−0.004 (2)
C11	0.053 (3)	0.039 (3)	0.075 (4)	0.004 (3)	−0.003 (3)	−0.003 (3)
C12	0.067 (4)	0.062 (4)	0.077 (5)	0.009 (3)	0.012 (4)	−0.004 (3)
C13	0.080 (6)	0.083 (5)	0.105 (7)	0.018 (4)	0.027 (5)	−0.005 (5)
C14	0.058 (5)	0.098 (7)	0.159 (11)	0.023 (5)	−0.003 (5)	−0.016 (6)
C15	0.080 (6)	0.145 (9)	0.136 (9)	0.039 (6)	−0.049 (6)	−0.053 (8)
C16	0.069 (5)	0.101 (7)	0.089 (6)	0.023 (5)	−0.017 (4)	−0.027 (5)
C17	0.052 (3)	0.041 (3)	0.062 (4)	−0.001 (2)	0.008 (3)	−0.005 (3)
C18	0.056 (4)	0.045 (3)	0.118 (6)	−0.008 (3)	0.026 (4)	−0.017 (4)
C19	0.063 (5)	0.157 (10)	0.101 (7)	0.010 (6)	0.004 (5)	0.006 (7)
C20	0.061 (6)	0.185 (14)	0.162 (11)	0.005 (7)	0.005 (6)	0.013 (11)
C21	0.075 (6)	0.105 (8)	0.186 (14)	−0.025 (5)	0.031 (8)	−0.023 (8)
C22	0.097 (8)	0.146 (11)	0.153 (11)	−0.006 (8)	0.066 (8)	0.014 (9)
C23	0.083 (6)	0.148 (10)	0.101 (7)	−0.014 (7)	0.024 (5)	0.011 (7)
C24	0.151 (8)	0.116 (7)	0.133 (8)	0.019 (7)	−0.008 (7)	−0.009 (7)

### Geometric parameters (Å, °)

**Table:**

Sn1—C1	2.142 (7)	C7—C8	1.368 (9)
Sn1—N1	2.250 (5)	C7—H7	0.9300
Sn1—N2	2.221 (5)	C8—C9	1.371 (8)
Sn1—S1	2.475 (2)	C8—H8	0.9300
Sn1—Cl1	2.515 (2)	C9—C10	1.479 (8)
Sn1—Cl2	2.496 (2)	C10—C11	1.480 (7)
Cl3—C24	1.715 (8)	C11—C12	1.3900
Cl4—C24	1.704 (8)	C11—C16	1.3900
Cl5—C24	1.795 (8)	C12—C13	1.3900
Cl3'—C24	1.703 (10)	C12—H12	0.9300
Cl4'—C24	1.755 (9)	C13—C14	1.3900
Cl5'—C24	1.707 (10)	C13—H13	0.9300
S1—C17	1.738 (6)	C14—C15	1.3900
N1—C5	1.336 (8)	C14—H14	0.9300
N1—C9	1.350 (8)	C15—C16	1.3900
N2—C10	1.313 (7)	C15—H15	0.9300
N2—N3	1.351 (7)	C16—H16	0.9300
N3—C17	1.338 (7)	C18—C23	1.489 (12)
N4—C17	1.337 (8)	C18—C19	1.527 (8)
N4—C18	1.456 (8)	C18—H18	0.9800
N4—H4	0.8600	C19—C20	1.525 (8)
C1—C2	1.474 (8)	C19—H19A	0.9700
C1—H1A	0.9700	C19—H19B	0.9700
C1—H1B	0.9700	C20—C21	1.507 (9)
C2—C3	1.475 (8)	C20—H20A	0.9700
C2—H2A	0.9700	C20—H20B	0.9700
C2—H2B	0.9700	C21—C22	1.510 (9)
C3—C4	1.478 (9)	C21—H21A	0.9700
C3—H3A	0.9700	C21—H21B	0.9700
C3—H3B	0.9700	C22—C23	1.540 (8)
C4—H4A	0.9600	C22—H22A	0.9700
C4—H4B	0.9600	C22—H22B	0.9700
C4—H4C	0.9600	C23—H23A	0.9700
C5—C6	1.364 (10)	C23—H23B	0.9700
C5—H5	0.9300	C24—H24	0.9800
C6—C7	1.367 (11)	C24—H24'	0.9801
C6—H6	0.9300		
			
C1—Sn1—N2	174.1 (2)	C15—C14—C13	120.0
C1—Sn1—N1	101.4 (2)	C15—C14—H14	120.0
N2—Sn1—N1	72.61 (18)	C13—C14—H14	120.0
C1—Sn1—S1	107.3 (2)	C14—C15—C16	120.0
N2—Sn1—S1	78.67 (13)	C14—C15—H15	120.0
N1—Sn1—S1	151.28 (13)	C16—C15—H15	120.0
C1—Sn1—Cl2	93.2 (2)	C15—C16—C11	120.0
N2—Sn1—Cl2	86.17 (13)	C15—C16—H16	120.0
N1—Sn1—Cl2	85.49 (14)	C11—C16—H16	120.0
S1—Sn1—Cl2	93.38 (6)	N4—C17—N3	116.1 (5)
C1—Sn1—Cl1	95.0 (3)	N4—C17—S1	115.5 (4)
N2—Sn1—Cl1	84.68 (13)	N3—C17—S1	128.4 (5)
N1—Sn1—Cl1	83.71 (14)	N4—C18—C23	111.0 (6)
S1—Sn1—Cl1	93.11 (6)	N4—C18—C19	109.1 (6)
Cl2—Sn1—Cl1	167.54 (7)	C23—C18—C19	110.1 (7)
C17—S1—Sn1	95.6 (2)	N4—C18—H18	108.9
C5—N1—C9	119.3 (5)	C23—C18—H18	108.9
C5—N1—Sn1	124.4 (5)	C19—C18—H18	108.9
C9—N1—Sn1	116.1 (4)	C20—C19—C18	111.0 (6)
C10—N2—N3	119.1 (5)	C20—C19—H19A	109.4
C10—N2—Sn1	118.7 (4)	C18—C19—H19A	109.4
N3—N2—Sn1	122.2 (4)	C20—C19—H19B	109.4
C17—N3—N2	114.5 (5)	C18—C19—H19B	109.4
C17—N4—C18	125.8 (5)	H19A—C19—H19B	108.0
C17—N4—H4	117.1	C21—C20—C19	111.6 (7)
C18—N4—H4	117.1	C21—C20—H20A	109.3
C2—C1—Sn1	115.1 (6)	C19—C20—H20A	109.3
C2—C1—H1A	108.5	C21—C20—H20B	109.3
Sn1—C1—H1A	108.5	C19—C20—H20B	109.3
C2—C1—H1B	108.5	H20A—C20—H20B	108.0
Sn1—C1—H1B	108.5	C22—C21—C20	112.5 (7)
H1A—C1—H1B	107.5	C22—C21—H21A	109.1
C1—C2—C3	119.8 (7)	C20—C21—H21A	109.1
C1—C2—H2A	107.4	C22—C21—H21B	109.1
C3—C2—H2A	107.4	C20—C21—H21B	109.1
C1—C2—H2B	107.4	H21A—C21—H21B	107.8
C3—C2—H2B	107.4	C21—C22—C23	110.8 (7)
H2A—C2—H2B	106.9	C21—C22—H22A	109.5
C4—C3—C2	120.8 (9)	C23—C22—H22A	109.5
C4—C3—H3A	107.1	C21—C22—H22B	109.5
C2—C3—H3A	107.1	C23—C22—H22B	109.5
C4—C3—H3B	107.1	H22A—C22—H22B	108.1
C2—C3—H3B	107.1	C18—C23—C22	112.1 (8)
H3A—C3—H3B	106.8	C18—C23—H23A	109.2
C3—C4—H4A	109.5	C22—C23—H23A	109.2
C3—C4—H4B	109.5	C18—C23—H23B	109.2
H4A—C4—H4B	109.5	C22—C23—H23B	109.2
C3—C4—H4C	109.5	H23A—C23—H23B	107.9
H4A—C4—H4C	109.5	Cl5'—C24—Cl4	34.0 (7)
H4B—C4—H4C	109.5	Cl5'—C24—Cl3'	109.4 (8)
N1—C5—C6	121.8 (7)	Cl4—C24—Cl3'	125.5 (11)
N1—C5—H5	119.1	Cl5'—C24—Cl3	121.9 (12)
C6—C5—H5	119.1	Cl4—C24—Cl3	111.8 (7)
C7—C6—C5	119.3 (7)	Cl3'—C24—Cl3	41.0 (7)
C7—C6—H6	120.3	Cl5'—C24—Cl4'	106.7 (7)
C5—C6—H6	120.3	Cl4—C24—Cl4'	73.3 (7)
C6—C7—C8	119.1 (6)	Cl3'—C24—Cl4'	106.0 (7)
C6—C7—H7	120.4	Cl3—C24—Cl4'	65.1 (7)
C8—C7—H7	120.4	Cl5'—C24—Cl5	69.3 (7)
C9—C8—C7	119.8 (7)	Cl4—C24—Cl5	103.1 (6)
C9—C8—H8	120.1	Cl3'—C24—Cl5	62.1 (7)
C7—C8—H8	120.1	Cl3—C24—Cl5	102.5 (6)
N1—C9—C8	120.6 (6)	Cl4'—C24—Cl5	163.2 (12)
N1—C9—C10	116.1 (5)	Cl5'—C24—H24	123.4
C8—C9—C10	123.3 (6)	Cl4—C24—H24	112.9
N2—C10—C11	123.3 (5)	Cl3'—C24—H24	121.2
N2—C10—C9	116.0 (5)	Cl3—C24—H24	112.9
C11—C10—C9	120.7 (5)	Cl4'—C24—H24	83.2
C12—C11—C16	120.0	Cl5—C24—H24	112.9
C12—C11—C10	120.1 (4)	Cl5'—C24—H24'	112.5
C16—C11—C10	119.9 (4)	Cl4—C24—H24'	120.3
C13—C12—C11	120.0	Cl3'—C24—H24'	110.5
C13—C12—H12	120.0	Cl3—C24—H24'	124.2
C11—C12—H12	120.0	Cl4'—C24—H24'	111.5
C12—C13—C14	120.0	Cl5—C24—H24'	84.7
C12—C13—H13	120.0	H24—C24—H24'	28.2
C14—C13—H13	120.0		
			
C1—Sn1—S1—C17	−174.4 (3)	C5—N1—C9—C10	179.0 (6)
N2—Sn1—S1—C17	5.4 (2)	Sn1—N1—C9—C10	−6.7 (7)
N1—Sn1—S1—C17	6.8 (4)	C7—C8—C9—N1	2.3 (10)
Cl2—Sn1—S1—C17	−80.0 (2)	C7—C8—C9—C10	−178.5 (6)
Cl1—Sn1—S1—C17	89.4 (2)	N3—N2—C10—C11	3.3 (9)
C1—Sn1—N1—C5	0.0 (6)	Sn1—N2—C10—C11	−176.8 (4)
N2—Sn1—N1—C5	−179.7 (6)	N3—N2—C10—C9	−176.4 (5)
S1—Sn1—N1—C5	178.9 (4)	Sn1—N2—C10—C9	3.5 (7)
Cl2—Sn1—N1—C5	−92.3 (5)	N1—C9—C10—N2	2.2 (8)
Cl1—Sn1—N1—C5	93.9 (5)	C8—C9—C10—N2	−177.0 (6)
C1—Sn1—N1—C9	−174.0 (5)	N1—C9—C10—C11	−177.5 (5)
N2—Sn1—N1—C9	6.2 (4)	C8—C9—C10—C11	3.3 (9)
S1—Sn1—N1—C9	4.8 (6)	N2—C10—C11—C12	−117.1 (6)
Cl2—Sn1—N1—C9	93.6 (4)	C9—C10—C11—C12	62.6 (7)
Cl1—Sn1—N1—C9	−80.1 (4)	N2—C10—C11—C16	63.1 (7)
C1—Sn1—N2—C10	−8(3)	C9—C10—C11—C16	−117.3 (5)
N1—Sn1—N2—C10	−5.2 (4)	C16—C11—C12—C13	0.0
S1—Sn1—N2—C10	174.1 (5)	C10—C11—C12—C13	−179.8 (5)
Cl2—Sn1—N2—C10	−91.7 (4)	C11—C12—C13—C14	0.0
Cl1—Sn1—N2—C10	79.9 (4)	C12—C13—C14—C15	0.0
C1—Sn1—N2—N3	172 (2)	C13—C14—C15—C16	0.0
N1—Sn1—N2—N3	174.7 (5)	C14—C15—C16—C11	0.0
S1—Sn1—N2—N3	−6.0 (4)	C12—C11—C16—C15	0.0
Cl2—Sn1—N2—N3	88.3 (4)	C10—C11—C16—C15	179.8 (5)
Cl1—Sn1—N2—N3	−100.2 (4)	C18—N4—C17—N3	6.0 (11)
C10—N2—N3—C17	−176.7 (6)	C18—N4—C17—S1	−174.4 (6)
Sn1—N2—N3—C17	3.4 (7)	N2—N3—C17—N4	−176.7 (6)
N2—Sn1—C1—C2	154 (2)	N2—N3—C17—S1	3.7 (9)
N1—Sn1—C1—C2	151.1 (9)	Sn1—S1—C17—N4	173.2 (5)
S1—Sn1—C1—C2	−28.3 (9)	Sn1—S1—C17—N3	−7.3 (6)
Cl2—Sn1—C1—C2	−122.8 (9)	C17—N4—C18—C23	117.7 (9)
Cl1—Sn1—C1—C2	66.6 (9)	C17—N4—C18—C19	−120.8 (9)
Sn1—C1—C2—C3	178.8 (11)	N4—C18—C19—C20	−179.1 (8)
C1—C2—C3—C4	13 (3)	C23—C18—C19—C20	−57.1 (11)
C9—N1—C5—C6	−0.2 (11)	C18—C19—C20—C21	55.1 (13)
Sn1—N1—C5—C6	−174.0 (6)	C19—C20—C21—C22	−53.4 (14)
N1—C5—C6—C7	1.6 (12)	C20—C21—C22—C23	52.4 (14)
C5—C6—C7—C8	−1.0 (12)	N4—C18—C23—C22	178.1 (8)
C6—C7—C8—C9	−0.9 (11)	C19—C18—C23—C22	57.2 (11)
C5—N1—C9—C8	−1.8 (9)	C21—C22—C23—C18	−55.0 (13)
Sn1—N1—C9—C8	172.6 (5)		

### Hydrogen-bond geometry (Å, °)

**Table:**

*D*—H···*A*	*D*—H	H···*A*	*D*···*A*	*D*—H···*A*
N4—H4···Cl1^i^	0.86	2.54	3.383 (6)	167

Symmetry codes: (i) −*x*+3/2, *y*+1/2, −*z*+1/2.
